# Reprogramming non-human primate somatic cells into functional neuronal cells by defined factors

**DOI:** 10.1186/1756-6606-7-24

**Published:** 2014-04-03

**Authors:** Zhi Zhou, Kazuhisa Kohda, Keiji Ibata, Jun Kohyama, Wado Akamatsu, Michisuke Yuzaki, Hirotaka James Okano, Erika Sasaki, Hideyuki Okano

**Affiliations:** 1 Department of Physiology, Keio University School of Medicine, 35 Shinanomachi, Shinjuku-ku, Tokyo 160-8582, Japan; 2 Division of Regenerative Medicine, Jikei University School of Medicine, 3-25-8 Nishi-Shinbashi, Minato-ku, Tokyo 105-8461, Japan; 3 Center of Applied Developmental Biology, Central Institute for Experimental Animals, 3-25-12 Tonomachi, Kawasaki-ku, Kawasaki, Kanagawa 210-0821, Japan

**Keywords:** Common marmoset, Direct reprogramming, Induced neuronal cells, Transcription factor, Regenerative medicine, Disease modeling, Cell-fate plasticity, Transdifferentiation

## Abstract

**Background:**

The common marmoset (*Callithrix jacchus*) is a New World primate sharing many similarities with humans. Recently developed technology for generating transgenic marmosets has opened new avenues for faithful recapitulation of human diseases, which could not be achieved in rodent models. However, the longer lifespan of common marmosets compared with rodents may result in an extended period for *in vivo* analysis of common marmoset disease models. Therefore, establishing rapid and efficient techniques for obtaining neuronal cells from transgenic individuals that enable *in vitro* analysis of molecular mechanisms underlying diseases are required. Recently, several groups have reported on methods, termed direct reprogramming, to generate neuronal cells by defined factors from somatic cells of various kinds of species, including mouse and human. The aim of the present study was to determine whether direct reprogramming technology was applicable to common marmosets.

**Results:**

Common marmoset induced neuronal (cjiN) cells with neuronal morphology were generated from common marmoset embryonic skin fibroblasts (cjF) by overexpressing the neuronal transcription factors: *ASCL1*, *BRN2*, *MYT1L* and *NEUROD1*. Reverse transcription-polymerase chain reaction of cjiN cells showed upregulation of neuronal genes highly related to neuronal differentiation and function. The presence of neuronal marker proteins was also confirmed by immunocytochemistry. Electrical field stimulation to cjiN cells increased the intracellular calcium level, which was reversibly blocked by the voltage-gated sodium channel blocker, tetrodotoxin, indicating that these cells were functional. The neuronal function of these cells was further confirmed by electrophysiological analyses showing that action potentials could be elicited by membrane depolarization in current-clamp mode while both fast-activating and inactivating sodium currents and outward currents were observed in voltage-clamp mode. The 5-bromodeoxyuridine (BrdU) incorporation assay showed that cjiN cells were directly converted from cjFs without passing a proliferative state.

**Conclusions:**

Functional common marmoset neuronal cells can be obtained directly from embryonic fibroblasts by overexpressing four neuronal transcription factors under *in vitro* conditions. Overall, direct conversion technology on marmoset somatic cells provides the opportunity to analyze and screen phenotypes of genetically-modified common marmosets.

## Background

The common marmoset (*Callithrix jacchus*) is a New World primate that has recently attracted considerable attention as a non-human primate model for biomedical research [1]. Specific features of the common marmoset are its small size, ease of handling, high fertility, early sexual maturity, its similarity of physiological properties with humans, drug metabolism, and neurophysiological functions [1]. Thus far, transgenic mice modeling human neurodegenerative diseases have contributed to disease research and drug development. However, none of them have succeeded in faithfully recapitulating the full spectrum of disease pathologies observed in humans [2,3]. In a recent report by our group, transgenic marmosets with germline transmission were successfully generated for the first time by lentiviral vector-mediated gene transfer [4]. For these reasons, our novel transgenic non-human primate models may be suitable for studying human diseases, particularly those that are neurodegenerative, such as Alzheimer’s and Parkinson’s disease. These *in vivo* models are expected to faithfully recapitulate pathophysiology of human diseases, and thus provide for the missing link between mouse and human disease research with subsequent drug development. However, results of studies from these models may require an extended period because of the longer lifespan of common marmosets compared with mice [5]. Moreover, detailed *in vitro* analyses using primary neuronal cultures of the affected area of the common marmoset transgenic models are not realistic.

Recent studies using human neuronal cells derived from either pluripotent stem cells or somatic cells have succeeded in modeling human neurological disorders *in vitro*[6,7]. These results prompted us to develop a convenient and rapid method for obtaining common marmoset neuronal cells from accessible somatic cells. Therefore, we focused on somatic cell reprogramming technology, including induced pluripotent stem (iPS) cell and direct conversion technology [8,9]. However, few studies have succeeded in generating common marmoset iPS cells from neonatal skin fibroblasts, fetal liver cells, and adult bone marrow-derived cells [10-12]. Moreover, only a few protocols exist for obtaining functional common marmoset neuronal cells from pluripotent stem cells [13]. Furthermore, little attention has been given to the direct conversion technology of common marmoset dermal fibroblasts into neuronal cells thus far. Therefore, in the present study, we aimed to generate common marmoset neuronal cells directly from dermal fibroblasts. Our results provide the first line of evidence for the generation of electrophysiologically functional neuronal cells from common marmoset somatic cells by defined neuronal transcription factors.

## Results and discussion

### Validation of the lentivirus-mediated overexpression of neuronal transcription factors

Recently, generation of induced neuronal (iN) cells was reported using mouse and human dermal cells [9,14]. In the present study, we used a set of neuronal transcription factors for human iN cells on common marmoset embryonic skin fibroblasts (cjF) isolated from embryonic day 91 (E91) embryos to determine whether common marmoset somatic cells could be converted into neuronal cells. We first confirmed transgene expression in the mouse fibroblast cell line, NIH3T3, by infecting these cells with lentiviral vectors coding the neuronal transcription factors: *ASCL1*, *BRN2*, *MYT1L*, and *NEUROD1*[14], under the control of tetracycline response element (TRE) together with reverse tetracycline transactivator (rtTA)-expressing vector. Upregulation of transgenes in NIH3T3 cells after doxycycline (dox) treatment was confirmed by immunocytochemistry (Additional file 1).

### Generation of neuron-like cells from common marmoset somatic cells

**Figure 1:**
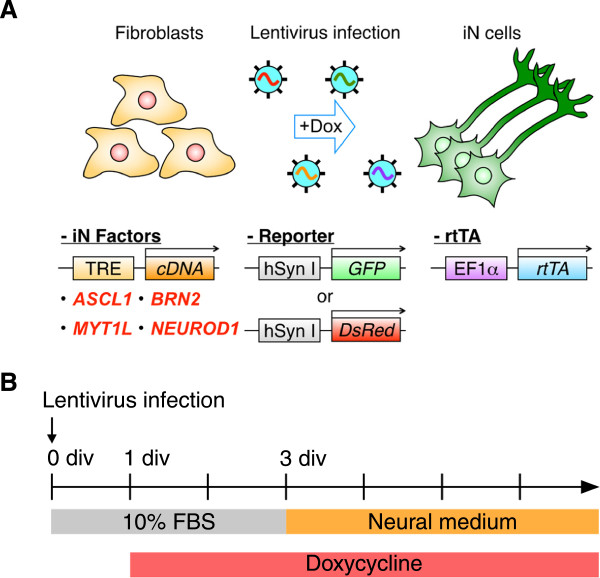
**Schematic overview of the experimental procedure for generation of common marmoset induced neuronal cells from common marmoset embryonic skin fibroblasts. (A)** Common marmoset embryonic skin fibroblasts (cjFs) were infected with drug-inducible lentiviral vectors coding a set of induced neuronal (iN) cell factors: *ASCL1*, *BRN2*, *MYT1L*, and *NEUROD1*. Synapsin reporter lentivirus expressing green fluorescent protein (GFP) or DsRed under the human synapsin I promoter was used to monitor neuronal induction. The lentiviral vector that stably drives *rtTA* expression under the EF1α promoter was transduced to induce the expression of iN-factors. **(B)** Time course for common marmoset induced neuronal (cjiN) cell induction. Lentivirus infection was conducted at 0 day *in vitro* (div) in fibroblast medium containing 10% fetal bovine serum, and cells were exposed to doxycycline (dox) at 1 div for cjiN induction. The culture medium was then replaced at 3 div with dox-containing neural meium composed of N2B27 medium, neurotrophin-3 (NT-3) and brain-derived neurotrophic factor (BDNF). For calcium imaging, the cAMP analog, 8-(4-Chlorophenylthio) adenosine 3′, 5′-cyclic monophosphate (8-CPT; 100 μM) [29], was added to promote neuronal maturation and survival.

**Figure 2:**
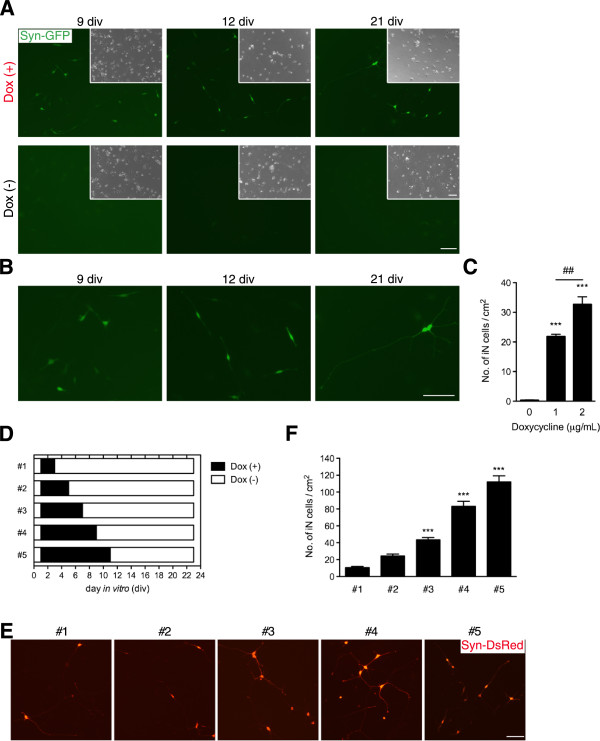
**Generation of iN cells. (A, B)** Transgene-dependent conversion of common marmoset fibroblasts into neuronal cells, in which synapsin reporter activation and morphological changes were dependent on the exogenous transcription factor and time. Cells became synapsin reporter-positive accompanied by a gradual morphological changes into neuronal cells, both effects of which were not found in cells cultured in neural medium without dox at 21 div. **(B)** Magnified images of Figure 1A showing synapsin reporter-positive cells with morphological changes from fibroblasts to neuronal cells. **(C)** Counts of synapsin reporter-positive cells with neuronal morphology showed a dox-dependent increase in their number (positive cells with fibroblast morphology were excluded from the counts). P^***^ < 0.001, *P*^##^ < 0.01 (one-way ANOVA followed by Tukey’s test). **(D-F)** The effect of sustained dox treatment on the production of cjiN cells. **(D)** Cells were treated with dox at 1 div until 3, 5, 7, 9 or 11 div and then maintained without dox until 23 div. **(E)** Synapsin reporter-positive cells with neuronal morphology were observed in all treatment group. **(F)** Cell counts revealed that a longer treatment time of dox increased the number of cjiN cells. Although dox treatment from 1–3 div sufficiently promoted cjiNs, a longer treatment time increased their number. P^***^ < 0.001 (one-way ANOVA followed by Tukey’s test). Scale bar; 200 μm.

To address whether cjFs, which were immunonegative for the neural progenitor marker, SRY (sex determining region Y)-box 2 (Sox2) (data not shown), can be converted into cjiN cells, cjFs were infected with these lentiviral vectors at 0 day *in vitro* (div) (Figure 1A). Synapsin reporter-positive mouse iN cells have been shown to be more functionally mature than negative cells [15]. Therefore, the reporter lentivirus, which expresses fluorescent protein (enhanced green fluorescent protein or DsRed) under the control of the human synapsin I promoter, was used in the present study to monitor neuronal conversion of cjFs [16-18] (Figure 1A). When cjFs were treated with dox at 1 div to induce neuronal conversion (Figure 1B), synapsin reporter-positive cells with typical neuronal morphologies were observed (Figure 2A, B). However, cjFs without dox treatment did not generate synapsin reporter-positive cells (Figure 2A). Notably, the morphology of synapsin reporter-positive cells resembled fibroblasts at 9 div and then changed into neuronal ones during reprogramming (Figure 2A, B). The reprogramming efficiency was monitored by the number of synapsin reporter-positive cells with neuronal morphology, and depended on the concentration of dox yielding 0.3 ± 0.1, 21.8 ± 0.8, or 32.6 ± 2.6 cjiN cells/cm^2^ at 16 div when treated with 0, 1, or 2 μg/mL dox, respectively (Figure 2C) (P^***^ < 0.001, *P*^##^ < 0.01, n = 4). However, the overall induction efficiency was much lower (<1%) than those in previous studies using mouse and human fibroblasts [9,14]. This discrepancy was probably due to the low infection efficiency of lentivirus in cjFs compared with those in mouse and human fibroblasts. Previous studies have also shown that polycistronic vector, micro RNAs and small molecules can facilitate iN cell induction [7,19,20]. Therefore, the induction conditions of cjiN cells may be optimized in the future study to enhance the conversion efficiency. Nevertheless, our results show that the four-factor set of neuronal transcription factors is sufficient to convert cjFs to cjiN cells.

### The duration of dox treatment was critical for neuronal conversion

Next, we investigated the required duration of exogenous neuronal transcription factor expression to drive neuronal transdifferentiation. Therefore, we examined the exposure time of dox and the cjiN induction efficiency (Figure 2D). Treatment with dox from 1–3 div promoted the conversion of cjFs into synapsin reporter-positive cells with neuronal morphology (Figure 2E). Similarly, a previous study has shown that the endogenous neuronal transcriptional factor network is activated 48 h after dox treatment [14]. Our findings also revealed that the induction efficiency at 23 div depended on the exposure time of dox (Figure 2F). The treatment of cjFs with dox from 1–11 div followed by a culture without dox treatment from 11–23 div generated 112.1 ± 28.1 cjiN cells/cm^2^ (Figure 2F). However, cells cultured with dox from 1–3 div followed by a culture without dox treatment from 3–23 div generated only 10.6 ± 5.4 cjiN cells/cm^2^ (Figure 2F). The efficiency of the former group was significantly (P^***^ < 0.001, n = 4) higher (more than 10-fold) compared with the latter group (Figure 2F). These results indicate that a longer expression of exogenous neuronal transcriptional factors is required for efficient lineage conversion of somatic cells.

### cjiN cells expressed a subset of neuronal genes and proteins

**Figure 3:**
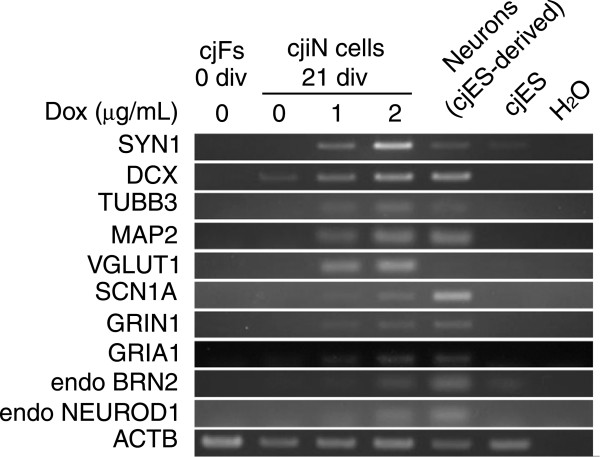
**Neuronal marker gene expression in cjiN cells.** Dox treatment upregulated cytoskeletal (*MAP2*, *DCX*, and *TUBB3*) and synaptic vesicular (*SYN1* and *VGLUT1*) marker genes, and cation channel-related genes (*SCN1A*, *GRIN1* and *GRIA1*). The results also showed the activation of endogenous *BRN2* and *NEUROD1* genes.

To characterize cjiN cells as neuronal cells, we performed reverse transcription-polymerase chain reaction (RT-PCR) analysis using bulk RNA samples, including the remaining synapsin reporter-negative cells to explore the expression of neuronal genes (see Table 1 for the primer sets used). We detected an upregulation of neuronal genes (Figure 3), which included cytoskeletal markers (*MAP2*, *DCX* and *TUBB3*), synaptic vesicle markers (*SYN1* and *VGLUT1*), and cation channel-related genes (*SCN1A*, *GRIN1* and *GRIA1*) in cjiN cells at 21 div (Figure 3). However, expression of these neuronal genes was not detected in cjFs at 0 div (Figure 3). These results were in line with the concentration-dependent induction efficiency of dox in Figure 2C. We also detected endogenous expression of *BRN2* and *NEUROD1* in cjiN cells at 21 div (Figure 3), indicating that ectopic expression of neuronal transcription factors activated the endogenous neuronal program. This effect may have caused neuronal transdifferentiation from somatic cells [9,14,21]. Thus, ectopic neuronal differentiation signals are likely to work together with the endogenous neuronal program to efficiently convert non-neuronal cells into neuronal cells [21].

**Table 1:** Primer sets used in RT-PCR analysis

**Gene name**	**Forward primer**	**Reverse primer**	**Product size (bp)**
SYN1	acggagactaccgcagtttg	cgatctgctccagcattgca	459
DCX	ctgtgcgtgtgcttctgaac	tcagctggagacttgcttcg	346
TUBB3	catagaccccagtggcaactacg	caccctccgtgtagtggcccttgg	241
MAP2	cctgtgttaagcggaaaacc	agagactttgtcctttgcctgt	86
VGLUT1	tcaataacagcacgacccac	tcccggaatttgagtgacaatg	131
SCN1A	attggcaattccgtgggagc	cccacacagcacgcggaaca	204
GRIN1	accccaagatcgtcaacatcg	ggctaaccagaatggcgtaga	213
GRIA1	cgagctttcctgttgatacat	tctgccacttgtaatggtcgatg	99
endo BRN2	aattaaggaaaaaggaaagcaact	caaaacatcattacacctgct	71
endo NEUROD1	gttattgtgttgccttagcacttc	agtgaaatgaattgctcaaattgt	77
ACTB	ggcatccacgaaactaccttt	acactgagtacttgcgctcg	202

**Figure 4:**
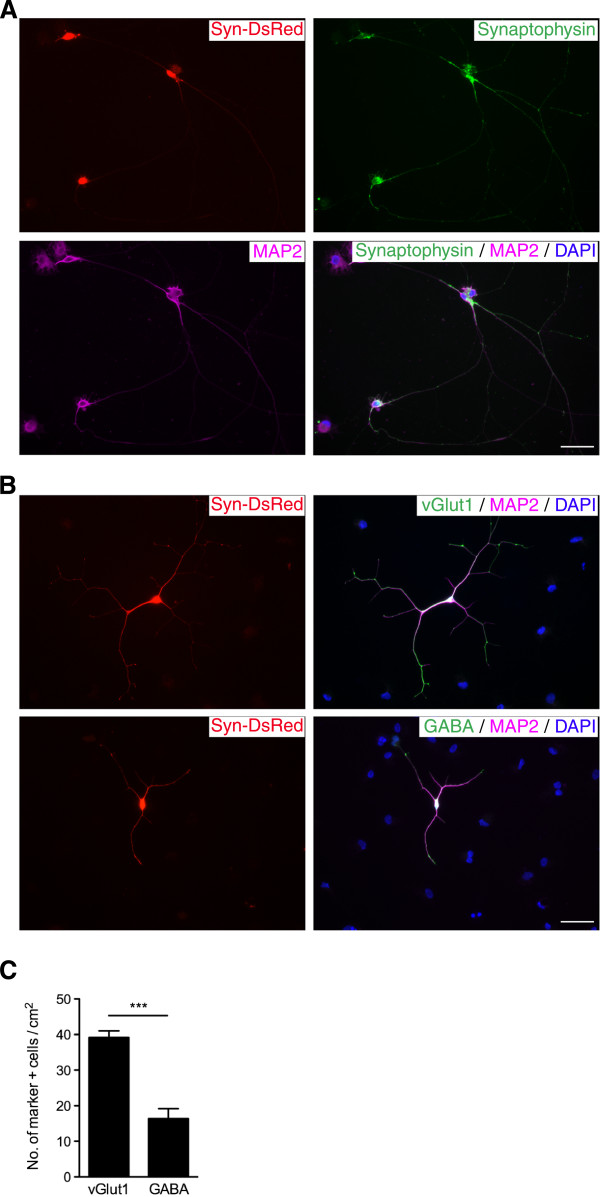
**Immunocytochemistry of cjiN cells. (A)** Synapsin reporter-positive-cjiN cells expressed microtubule-associated protein 2 (MAP2) and synaptophysin. **(B)** cjiN cells were vesicular glutamate transporter 1 (vGlut1)- and gamma-aminobutyric acid (GABA)-positive, thereby displaying a heterogeneous pool of excitatory and inhibitory neurons, respectively. **(C)** A significantly greater number of vGlut1-positive cells was generated compared with GABA-positive cells. P^***^ < 0.001 (Student’s *t*-test). Scale bar; 50 μm.

To further characterize the property of cjiN cells, we performed immunocytochemistry for the neuronal markers. The results showed that most synapsin reporter-positive cells also expressed (>88%) the pan-neuronal marker, microtubule-associated protein 2 (MAP2) (data not shown). This result indicated that the synapsin reporter using the sequence of human synapsin I promoter was also functional in common marmoset cells, and was thus a reliable reporter for neuronal conversion, as previously reported [15,16]. In cjiN cells at 43 div, MAP2-negative cells were found to be negative for glial fibrillary acidic protein and only weakly positive for α-smooth muscle actin (data not shown), raising the possibility that they were partially reprogrammed cells. The MAP2- and synapsin reporter-positive cells also showed immunoreactivity for the synaptic vesicle marker, synaptophysin, at 38 div (Figure 4A). However, immunostaining for synaptophysin and PSD95 revealed that these cells were unlikely to make synapse structures at 38 div (data not shown). Although the cjiN cells expressed neuronal marker genes and proteins, they did not appear to be mature enough to make synaptic contacts themselves. However, a future study may facilitate synaptic formation by improving induction efficiency and by co-culturing with astrocytes [9,14].

### The majority of cjiN cells were glutamatergic

To characterize the neurotransmitter phenotype of iN cells, we examined the expression of neurotransmitters in MAP2-positive cjiN cells. Immunostaining at 38 div for vesicular glutamate transporter 1 (vGlut1) and gamma aminobutyric acid (GABA) revealed the presence of both excitatory glutamatergic and inhibitory GABAergic neuronal cells, respectively (Figure 4B). Counts of vGlut1 and GABA-positive cjiN cells showed a significantly (P^***^ < 0.001, n = 4) greater number of vGlut1-positive cells (39.2 ± 1.9/cm^2^) than GABA-positive cells (16.4 ± 2.8 cells/cm^2^) (Figure 4C). These results are in accordance with previous studies showing that the majority of iN cells are excitatory cells [7,14,22]. Our findings thus indicate that iN induction may be feasible in the future for the *in vitro* analysis of the transgenic common marmoset model of Alzheimer’s disease, in which forebrain excitatory neurons are expected to be affected. Moreover, our cjiN cell induction protocol is likely advantageous over the previously reported neuronal differentiation protocol which used common marmoset embryonic stem (ES) cells and iPS cells, because the ES/iPS cell-derived neural precursor cells showed caudal identity [13]. Thus far, several groups have succeeded in generating reprogrammed neuronal cells with specific neuronal subtypes, such as dopaminergic neurons and motor neurons [23-26], which implicate the cell fate plasticity of terminally differentiated somatic cells. Our success in reprogramming common marmoset somatic cells into excitatory and inhibitory neuronal cells using defined iN factors may therefore provide great promise in the future for generating specific subtypes of neuronal cells with specific sets of neuronal transcription factors.

### cjiN cells were functional as matured neurons

**Figure 5:**
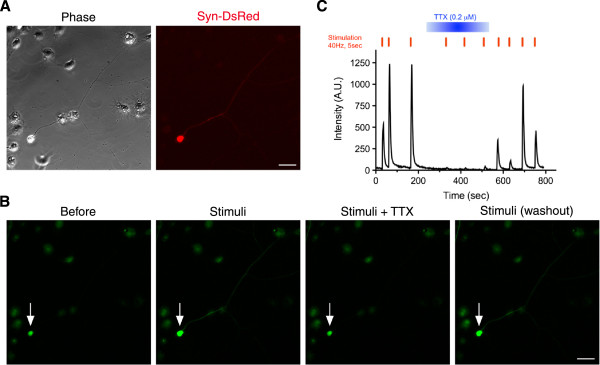
**Increase in the intracellular calcium level upon electrical field stimulation. (A)** cjiN cells (synapsin reporter-positive with a neuronal morphology). **(B, C)** Intracellular calcium level ([Ca^2+^]_i_) in cjiN cells was measured by the intensity of Fluo-4 fluorescence. Electrical field stimulation (40 Hz for 5 seconds) induces a robust elevation of [Ca^2+^]_i_ in some synapsin reporter-positive cells. The increase in [Ca^2+^]_i_ was reversibly suppressed by the specific voltage-gated sodium channel blocker, tetrodotoxin. Scale bar; 50 μm.

**Figure 6:**
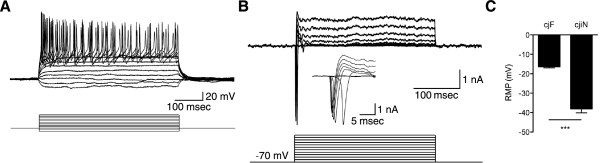
**Neuronal electrophysiological property of cjiN cells. (A)** cjiN cells elicited repetitive action potential by membrane depolarization in current-clamp mode. **(B)** cjiN cells showed both fast-activating and inactivating sodium currents and outward currents in voltage-clamp mode. **(C)** cjiN cells exhibited significantly lower resting membrane potentials than cjFs. P^***^ < 0.001 (Mann-Whitney *U*-test).

To further confirm the successful conversion of cjFs into functional neuronal cells, we performed calcium imaging analysis. cjiN cells cultured with dox were incubated with the calcium indicator, Fluo-4 AM [13], followed by response recordings. The intracellular calcium level ([Ca^2+^]_i_) in cjiN cells at 15 div was increased in cjiN cells perfused with 80 mM of KCl, which was then decreased by washout (Additional file 2). Furthermore, electrical field stimulation on cjiN cells at 28 div increased [Ca^2+^]_i_, which was reversibly blocked by the voltage-gated sodium channel blocker, tetrodotoxin (0.2 μM) (Figure 5), suggesting that the increase in [Ca^2+^]_i_ was likely evoked by action potentials through voltage-gated sodium channels. These results showed that cjiN cells derived from cjFs were functionally comparable to common marmoset ES cell-derived neuronal cells [13], and thus strongly suggest that reprogramming of common marmoset somatic cells generates functional neuronal cells. Moreover, electrophysiological analyses of cjiN cells at 29–42 div revealed that action potentials were elicited by membrane depolarization in current-clamp mode in 17 out of 21 cjiN cells (81.0%) (Figure 6A and Additional file 3). Among these 17 cjiN cells, 7 cjiN cells (41.2%) generated a single action potential and the remaining 10 cjiN cells (58.8%) generated repetitive action potentials (Figure 6A and Additional file 3). In the voltage-clamp mode, both fast-activating and inactivating sodium currents and outward currents were observed (Figure 6B). The resting membrane potentials of cjiN cells ranged between -26 and -55 mV, with a mean ± SEM of -38.1 ± 2.0 mV (Figure 6C and Additional file 3). This mean value was significantly (P^***^ < 0.001) lower than that of cjFs (-16.4 ± 0.6 mV) with a range between -13.1 and -19.0 mV (Figure 6C and Additional file 3). Overall, our patch clamp recordings showed that cjiN cells are functional neuronal cells.

### cjiN cells were directly converted from cjFs without passing through a proliferative state

**Figure 7:**
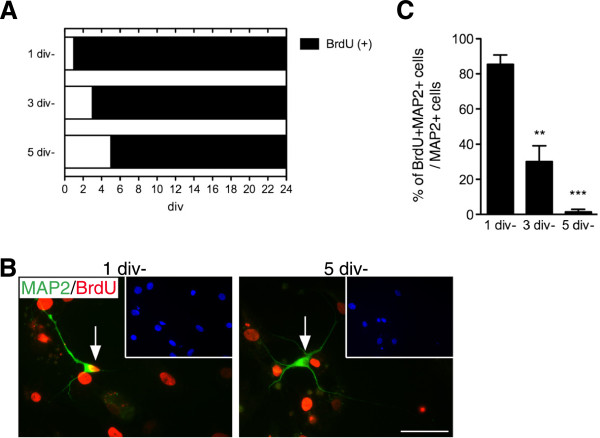
**Cells destined to be cjiN cells became postmitotic at the early phase of the reprogramming process. (A)** Time course for the BrdU incorporation experiment. BrdU (10 μM) was added into the culture medium at 1, 3 or 5 div until cells were fixed at 24 div. **(B, C)** Immunocytochemistry against MAP2 and BrdU revealed that when cells were treated with BrdU at 1 div until 24 div, 85.2 ± 5.4% of cjiN cells were BrdU + MAP2+, while when treated at 3 or 5 div until 24 div, only 30.1 ± 9.0% or 1.5 ± 1.5% of cjiN cells were BrdU + MAP2+, respectively, showing that the cjFs became postmitotic soon after transgene expression. P^***^ < 0.001 P^**^ < 0.01. Scale bar; 50 μm.

To determine whether cjiN cells were directly converted from cjFs without passing through a proliferative neural progenitor-like cell state, 5-bromodeoxyuridine (BrdU; 10 μM) was added to the media at 1, 3 or 5 div until 24 div (Figure 7A), and the percentage of double-positive cells for BrdU and MAP2 among the MAP2 single-positive cells was determined (Figure 7B, C). The result showed that while 85.3 ± 5.4% of MAP2-positive cells incorporated BrdU when treated from 1–24 div, only 30.1 ± 9.0% (P^**^ < 0.01) and 1.5 ± 1.5% (P^***^ < 0.001) of MAP2-positive cells incorporated BrdU when treated from 3–24 and 5–24 div, respectively (Figure 7C). This result indicates that most of the cells that were destined to be cjiN cells became postmitotic at the early phase of the reprogramming process, suggesting that cjiN induction is a direct process unless cells pass through a proliferative neural progenitor-like cell state, from which neuronal cells can be differentiated. In the present study, however, no Sox2-positive cells were found during 2–5 div, while MAP2-positive cells were present at 21 div (data not shown), indicating that the induction of neural progenitor-like cells is unlikely during 1-5 div. Therefore, these results show that cjiN cells are directly converted from cjFs without passing through proliferative neural progenitor cells.

## Conclusions

In the present study, we established an *in vitro* method to convert common marmoset somatic cells into functional neuronal (i.e. cjiN) cells. The majority of the cjiN cells were vGlut1-positive excitatory neuronal cells and expressed the neuronal marker genes: *TUBB3*, *DCX*, *MAP2*, *SYN1*, *VGLUT1*, *SCN1A*, *GRIN1* and *GRIA1*. Importantly, cjF-derived cjiN cells exhibited functional neuronal properties and responded to exogenous stimulation. Overall, these findings suggest that direct conversion technology may be beneficial in rapid and robust screening of neuronal phenotypes of transgenic common marmoset models of human diseases and analyzing underlying molecular mechanisms of diseases.

## Methods

### Animals

All animal experiments were approved by the Institutional Animal Care and Use Committee of the Central Institute for Experimental Animals (CIEA), and was performed in accordance with CIEA and Keio University guidelines.

### Cell culture

Common marmoset embryonic fibroblasts, NIH3T3 cells and 293T cells were cultured in Dulbecco’s modified Eagle’s medium supplemented with 10% fetal bovine serum, 100 U/mL of penicillin and 100 μg/mL of streptomycin (10% FP medium) at 37°C with 5% CO_2_ incubation.

### Molecular cloning and lentivirus production

cDNA entry clone of human *ASCL1* [GenBank: NM_004316] was purchased from DNAFORM (clone ID: 100006383, Japan). cDNAs of human *BRN2* [GenBank: NM_005604.3], *MYT1L* [GenBank: NM_015025.2] and *NEUROD1* [GenBank: NM_002500.4] were cloned into pENTR-D-TOPO vector (Invitrogen, USA). Then cDNAs were inserted into a self-inactivation human immunodeficiency virus-1-based lentivirus construct, CSIV-TRE-RfA (CSIV-TRE-RfA-CMV-KT was kindly provided by Dr. Hiroyuki Miyoshi (RIKEN BRC, Japan), and then modified by Dr. Takuji Maeda (Nagoya University, Japan)), by LR reaction (Invitrogen, USA). Similarly, reverse tetracycline transactivator (rtTA) gene was inserted into CSII-EF1α-RfA-TK-HygR construct [27]. The human synapsin I reporter constructs, pCSC-hSynI-GFP [16] and pHIV7-hSynI-DsRed [18], were kindly provided by Dr. Fred H. Gage, Salk Institute, USA, and Dr. Alysson R. Muotri, University of California, USA, respectively. Besides, CSIV-hSynI-GFP-IRES2-NeoR and CSIV-hSynI-DsRed-IRES2-NeoR were constructed in-house. These reporters were constructed using CSIV-TRE-RfA-CMV-KT, pCSC-hSynI-GFP, pHIV7-hSynI-DsRed and pIRESneo3 (Clontech, USA) with PCR- and restriction enzyme-based method. For lentivirus production, 293T cells were transfected with lentivirus plasmid, pCAG-HIVgp and pCMV-VSV-G-RSV-Rev [28] (kindly provided by Dr. Hiroyuki Miyoshi, RIKEN BRC, Japan). After 16-20 h, supernatant was replaced by fresh media followed by 48-72 h incubation. The virus containing media were then collected and 0.45 μm-filterated followed by ultracentrifugation. The concentrated virus was suspended in PBS and used in subsequent experiments.

### Induction of common marmoset iN cells

Common marmoset embryonic fibroblasts were seeded directly on culture ware at 1 × 10^4^ cells/cm^2^. Twenty-four hours later, the cells were infected with lentivirus in 10% FP media containing polybrene (8 μg/mL) (Sigma-Aldrich, USA). After 16–20 h in media containing lentivirus, the cells were switched into fresh 10% FP medium containing doxycycline (dox) (2 μg/mL) to drive transgene expression. Procedures for experiments determining the sufficient concentration and duration of dox are shown in the main text. After 48 h in 10% FP media with dox, the media was replaced with dox-containing neural media composed of N2B27 media [20], brain-derived neurotrophic factor (BDNF) (10 ng/mL, R&D systems, USA) and neurotrophin-3 (NT-3) (10 ng/mL, R&D systems, USA). For calcium imaging, 8-(4-Chlorophenylthio) adenosine 3′, 5′-cyclic monophosphate (8-CPT, 100 μM, Sigma-Aldrich, USA), one of the cAMP analogs [29], was also supplemented to promote neuronal maturation. The media was changed every 2–3 days during culture period. BrdU incorporation assay was performed as previously described [9] incubating cells with BrdU (10 μΜ, BD, USA) until cells were fixed.

### Immunocytochemistry

Cells were fixed with 4% paraformaldehyde for 15 minutes at room temperature and then processed for immunocytochemistry [13]. Samples were rinsed with PBS three times. Then, samples were incubated at 4°C overnight with the primary antibodies diluted in PBS containing 5% of fetal bovine serum and 0.3% Triton X-100. The primary antibodies used were as follows; Synaptophysin (1:50000, Millipore, USA), MAP2 (1:1000, Sigma-Aldrich, USA), MAP2 (1:500, Millipore, USA), vGlut1 (1:2000, Synaptic systems, Germany), GABA (1:1000, Sigma-Aldrich, USA), PSD95 (1:500, Millipore, USA), α-SMA (1:500, Sigma-Aldrich, USA), Sox2 (1:500, R&D, USA), Ascl1 (1:200, BD, USA), Brn2 (1:200, Santa Cruz, USA), Myt1L (1:500, Abcam, England) and NeuroD1 (1:500, Santa Cruz, USA). After three washes with PBS, samples were incubated with secondary antibodies conjugated with Alexa-488, Alexa-555 and Alexa-647 (Invitrogen, USA). Nuclei were counterstained with 4’, 6-diamidino-2-phenylindole (DAPI; 1:1000, Dojindo, Japan). After washing with PBS, samples were mounted on slides with FluorSave reagent (Calbiochem, Germany) and examined under a universal fluorescence microscope (Axioplan 2; Carl Zeiss, Germany). For anti-BrdU staining (1:500, Abcam, England), cells were treated with 1 N HCl in PBS for 30 minutes at 37°C and then rinsed with PBS three times before primary antibody incubation.

### RNA isolation and reverse transcription-polymerase chain reaction (RT-PCR)

Total RNA was isolated with RNeasy Micro Kit with DNase I treatment (QIAGEN, Germany) and was used to synthesize cDNA with ReverTraAce qPCR RT Kit (TOYOBO, Japan) according to the manufacturer’s instruction. RT-PCR was conducted using Ex Taq HS (TAKARA, Japan) according to the manufacturer’s instruction. Common marmoset ES (cjES) cells and cjES-derived neurons were used as control [13]. The primer sets used are listed in Table 1.

### Calcium imaging and electrical stimulation

Calcium imaging analyses were performed as described previously [13]. To load the calcium imaging dye, cells were incubated with 1 μM Fluo-4 AM (Invitrogen, USA) in imaging solution consisting of 117 mM NaCl, 2.5 mM KCl, 2 mM CaCl_2_, 2 mM MgSO_4_, 25 mM HEPES and 30 mM D-(+)-glucose, (pH 7.4), at 37°C for 20 minutes, followed by washing for 30 minutes in imaging solution. Coverslips were placed on a custom-made field stimulation chamber and mounted on the stage of a Nikon Eclipse microscope with a 20× (NA 0.45) objective. Cells were perfused at 2 ml/minute with the imaging solution at room temperature with or without 0.2 μM tetrodotoxin (TTX; Alomone Labs Ltd., Israel). Images were acquired at 2 Hz (500 millisecond exposure time) with a cooled CCD camera (Andor iXon, DU897). Extracellular field stimulation was performed with two parallel platinum wires at 25 V/cm. Each stimulation was a train of 500 microsecond pulses at 40 Hz for 5 seconds. Images were analyzed with ImageJ software (NIH, Bethesda, MD).

### Electrophysiology

Electrophysiological recordings were performed as described previously [30]. Synapsin reporter-positive cjiN cells were identified under an inverted microscope (Diaphot-TMD 200; Nikon, Japan) and whole cell patch clamp recordings were done using Axopatch 200B (Axon Instruments, USA) at room temperature. The extracellular solution composed of 117 mM NaCl, 2.5 mM KCl, 2 mM CaCl_2_, 2 mM MgCl_2_, 15 mM D-Glucose and 20 mM HEPES (pH 7.4 adjusted with NaOH, 304 mOsm) was continuously perfused during recordings. Patch pipettes had a resistance of 5-6 MΩ filled with the intracellular solution containing 130 mM K-gluconate, 1 mM CaCl_2_, 1 mM MgCl_2_, 10 mM EGTA, 10 mM Sucrose and 20 mM HEPES (pH adjusted with KOH, 305 mOsm). In voltage-clamp recordings, iN cells were held at -70 mV and voltage steps (10 mV, 300 msec) were applied to elicit voltage-activated currents. Action potentials were evoked by injecting step currents (20-40 pA, 500 msec) in the current-clamp mode. Data were digitized at 10 kHz with a 2 kHz low-pass filter. Liquid junction potential was corrected.

### Statistical analysis

All data were expressed as means ± SEM. The statistical significance of differences was analyzed by Student’s *t*-test, Mann-Whitney *U*-test or one-way ANOVA followed by Tukey’s test using Graph Pad Prism5 software. Differences of *P* < 0.05 were considered statistically significant.

## Abbreviations

cjiN cells: Common marmoset induced neuronal cells; cjF: Common marmoset embryonic fibroblast; RT-PCR: Reverse transcription-polymerase chain reaction; dox: Doxycycline; rtTA: Reverse tetracycline transactivator.

## Competing interests

H.O. is a paid scientific consultant to San Bio, Inc., Eisai Co., Ltd., and Daiichi Sankyo Co., Ltd.

## Authors’ contributions

ZZ conceived the concept of this study, designed the experiments, performed experiments and analyzed data. KI, KK and MY performed calcium imaging assays and electrophysiology, and ZZ, KI, KK and MY analyzed data. JK, WA, HJO and HO coordinated the study. ES prepared and provided common marmoset embryonic fibroblasts. HO provided financial support for the experiments. ZZ, JK and HO wrote the paper. All authors read and approved the final manuscript.

## Supplementary Material

Additional file 1 **Doxycycline-dependent transgene induction in NIH3T3 cells.** Immunocytochemistry against Ascl1, Brn2, Myt1L and NeuroD1 in a mouse fibroblast cell line, NIH3T3 cells, that were lentivirally transduced with iN factors and treated with doxycycline from 1–4 div revealed doxycycline-dependent transgene expressions. Scale bar; 100 μm. Click here for file

Additional file 2 **KCl perfusion increased the intracellular calcium level. (A)** cjiN cells (synapsin reporter-positive with a neuronal morphology). **(B, C)** Intracellular calcium level ([Ca^2+^]_i_) in cjiN cells was measured by the intensity of Fluo-4 fluorescence. KCl (80 mM) perfusion caused a robust elevation of [Ca^2+^]_i_ in some synapsin reporter-positive cells. This increase was reversibly suppressed by washout. Scale bar; 50 μm. Click here for file

Additional file 3 Electrophysiological parameters in cjiN cells at 29–42 div. Click here for file
