# Supplementary figures and images for: Reprogramming non-human primate somatic cells into functional neuronal cells by defined factors
Source: Mol Brain. 2014 Apr 3;7:24. doi: 10.1186/1756-6606-7-24 (PMC4021617; doi:10.1186/1756-6606-7-24)

Dox (+)

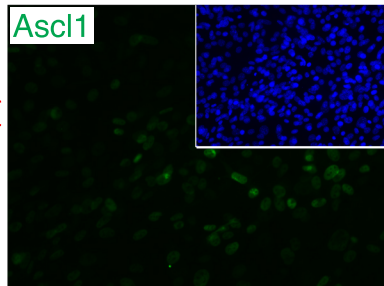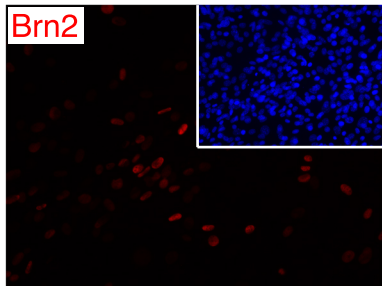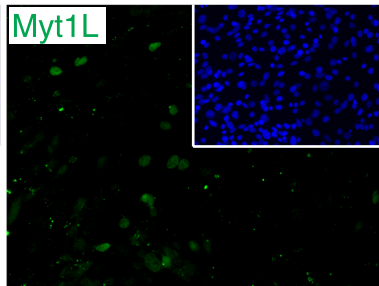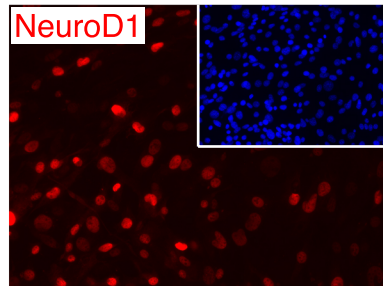

Dox (-)

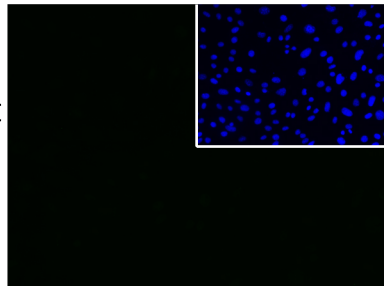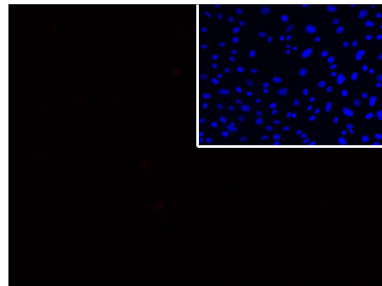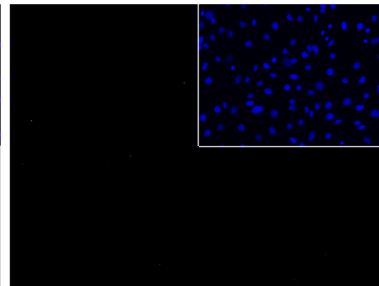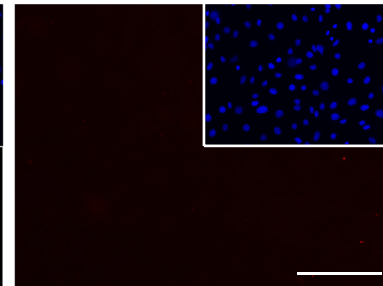

Supplement: Additional file 1 — Doxycycline-dependent transgene induction in NIH3T3 cells. Immunocytochemistry against Ascl1, Brn2, Myt1L and NeuroD1 in a mouse fibroblast cell line, NIH3T3 cells, that were lentivirally transduced with iN factors and treated with doxycycline from 1–4 div revealed doxycycline-dependent transgene expressions. Scale bar; 100 μm. [file 1756-6606-7-24-S1.pdf]

**A**

Phase

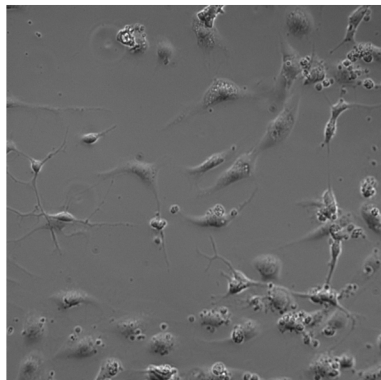

Syn-DsRed

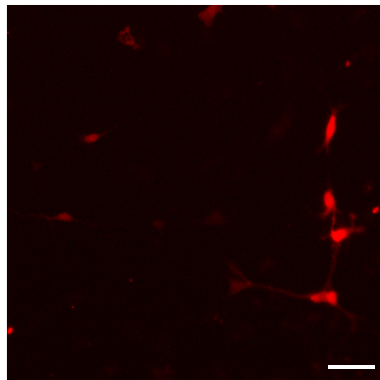**C**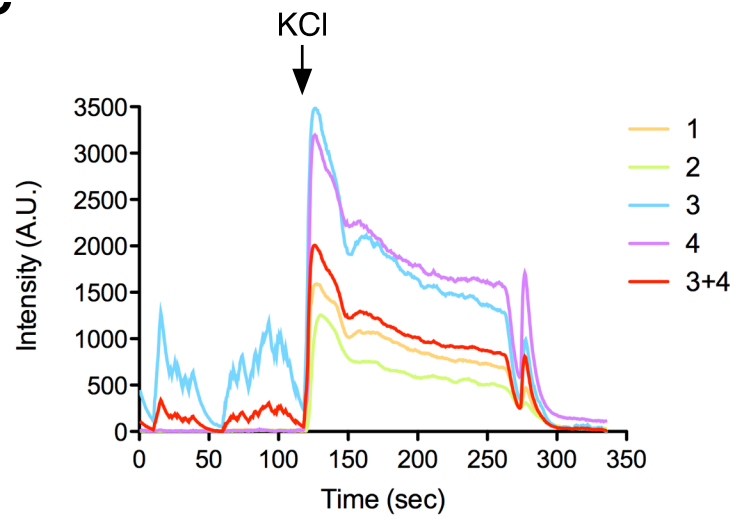**B**

Before

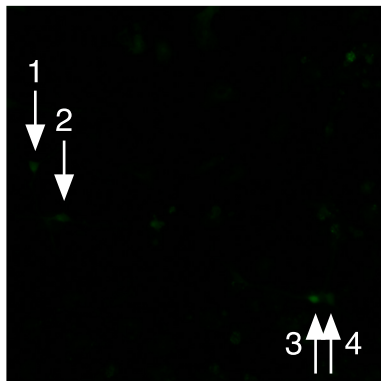

High KCl

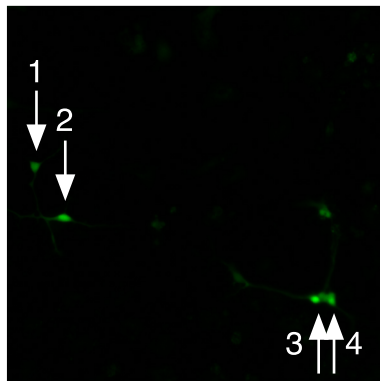

Washout

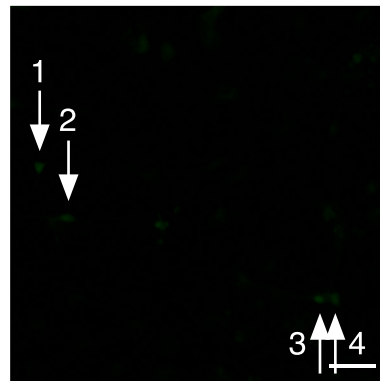

Supplement: Additional file 2 — KCl perfusion increased the intracellular calcium level. (A) cjiN cells (synapsin reporter-positive with a neuronal morphology). (B, C) Intracellular calcium level ([Ca2+]i) in cjiN cells was measured by the intensity of Fluo-4 fluorescence. KCl (80 mM) perfusion caused a robust elevation of [Ca2+]i in some synapsin reporter-positive cells. This increase was reversibly suppressed by washout. Scale bar; 50 μm. [file 1756-6606-7-24-S2.pdf]
