# Supplementary material for: Reprogramming non-human primate somatic cells into functional neuronal cells by defined factors
Source: Mol Brain. 2014 Apr 3;7:24. doi: 10.1186/1756-6606-7-24 (PMC4021617; doi:10.1186/1756-6606-7-24)
Supplement: Additional file 3 — Electrophysiological parameters in cjiN cells at 29–42 div. [file 1756-6606-7-24-S3.pdf]

Additional file 3.

cjiN cells

Cell No.	RMP (mV)	R_{in} (MΩ)	C_m (pF)	AP
1	-27.0	157.3	7.42	S
2	-31.0	100.9	11.90	S
3	-28.0	282.1	8.13	S
4	-35.0	2482.4	9.07	S
5	-30.5	713.4	4.58	N
6	-37.0	777.5	4.58	R
7	-46.0	512.4	7.65	N
8	-49.0	307.7	4.08	N
9	-46.0	821.8	4.96	N
10	-40.5	286.3	9.11	R
11	-43.1	270.2	20.84	R
12	-30.5	483.8	15.57	R
13	-26.0	318.5	14.36	R
14	-55.0	1667.0	19.44	R
15	-30.5	188.5	30.63	R
16	-39.6	221.3	6.33	S
17	-51.8	234.8	15.30	S
18	-35.0	299.0	6.59	S
19	-31.6	107.2	10.81	R
20	-53.0	132.8	18.85	R
21	-34.3	1599.6	9.39	R
Average	-38.1	569.7	11.41	
SEM	2.0	138.7	1.51	

RMP: resting membrane potential, R_{in}: membrane input resistance, C_m: capacitance, AP: action potential.

S: single, R: repetitive, N: not detected

cjFs

Cell No.	RMP (mV)	R_{in} (MΩ)	C_m (pF)
1	-19.0	681.6	10.63
2	-17.6	1227.4	9.26
3	-16.1	535.0	9.52
4	-18.7	1695.4	13.76
5	-17.8	2987.3	13.32
6	-13.4	319.2	10.51
7	-13.8	2516.5	22.65
8	-17.0	777.1	7.90
9	-13.1	571.3	9.35
10	-17.5	315.4	11.28
11	-17.7	599.4	7.58
12	-14.8	564.4	7.59
13	-16.8	958.8	12.17
Average	-16.4	1057.6	11.19
SEM	0.6	244.0	1.15

RMP: resting membrane potential, R_{in}: membrane input resistance, C_m: capacitance
